# Bupivacaine inhibits a small conductance calcium‐activated potassium type 2 channel in human embryonic kidney 293 cells

**DOI:** 10.1186/s40360-021-00481-2

**Published:** 2021-03-12

**Authors:** Hongfei Chen, Zhousheng Jin, Fangfang Xia, Zhijian Fu

**Affiliations:** 1 Department of Pain Management, Shandong Provincial Hospital, Cheeloo College of Medicine, Shandong University, 324 Jingwu Road, 250021 Jinan, China; 2 Department of Anesthesiology, The First Affiliated Hospital of Wenzhou Medical University, 325000 Wenzhou, Zhejiang, China

**Keywords:** Bupivacaine, Cardiotoxicity, HEK293 cells, Inhibition, SK2 channel

## Abstract

**Background:**

Bupivacaine blocks many ion channels in the heart muscle, causing severe cardiotoxicity. Small-conductance calcium-activated potassium type 2 channels (SK2 channels) are widely distributed in the heart cells and are involved in relevant physiological functions. However, whether bupivacaine can inhibit SK2 channels is still unclear. This study investigated the effect of bupivacaine on SK2 channels.

**Methods:**

The SK2 channel gene was transfected into human embryonic kidney 293 cells (HEK-293 cells) with Lipofectamine 2000. The whole-cell patch-clamp technique was used to examine the effect of bupivacaine on SK2 channels. The concentration–response relationship of bupivacaine for inhibiting SK2 currents (0 mV) was fitted to a Hill equation, and the half-maximal inhibitory concentration (IC50) value was determined.

**Results:**

Bupivacaine inhibited the SK2 channels reversibly in a dose-dependent manner. The IC50 value of bupivacaine, ropivacaine, and lidocaine on SK2 currents was 16.5, 46.5, and 77.8µM, respectively. The degree of SK2 current inhibition by bupivacaine depended on the intracellular concentration of free calcium.

**Conclusions:**

The results of this study suggested the inhibitory effect of bupivacaine on SK2 channels. Future studies should explore the effects of SK2 on bupivacaine cardiotoxicity.

## Background

Local anesthetics (LAs), such as bupivacaine, ropivacaine, and lidocaine, are often used for regional anesthesia and analgesia. Their cardiotoxicity also differs due to their different chemical structures: bupivacaine > ropivacaine > lidocaine. Bupivacaine is one of the long-acting, lipophilic LAs. It is used for analgesia perioperatively due to its high analgesic efficacy and long-lasting effect. However, accidental delivery or excessive absorption of bupivacaine into blood circulation may cause severe arrhythmia or even cardiac arrest [1–3]. Statistical estimates showed that the incidence of LA-induced toxicity in the peripheral nerve block was 7.5–20/10,000 [4, 5]. However, the mechanism of bupivacaine cardiotoxicity has not been fully elucidated. Bupivacaine can block sodium [6, 7], L-calcium [8, 9], and potassium channels [10, 11], which may be involved in bupivacaine cardiotoxicity.

Calcium-activated potassium channels are calcium-dependent channels triggered by intracellular calcium [12]. In humanṣ, calcium-activated potassium channels can be divided into three categories: large-conductance channels, intermediate-conductance channels, and small-conductance channels. Small-conductance calcium-activated potassium type 2 channels (SK2 channels) are involved in hyperpolarization after the action potential. These channels function in the atria [13], ventricles [13], atrioventricular nodes [14], and Purkinje cells [15], which play important roles in cardiac conduction. The dysfunction of SK2 channels may lead to atrial or ventricular arrhythmia due to the important role of these channels in regulating the action potential [3, 16]. SK2 protein expression and SK2 currents decreased in 22 patients with chronic atrial fibrillation compared with patients with sinus rhythm [17]. The effect of bupivacaine on SK2 channels was not reported thus far. It was hypothesized that bupivacaine directly suppressed SK2 currents.

In this study, HEK 293 cells were transfected with the SK2 gene. The whole-cell patch-clamp technique was used to demonstrate that bupivacaine could inhibit SK2 currents. The aim of the study was to demonstrate the ability of bupivacaine to inhibit SK2 channels and the effect of calcium concentration on its blockade.

## Methods

### Cell line culture and gene transfection

HEK293 cells were all purchased from the Institute of Life Sciences of the Chinese Academy of Sciences (China). After harvesting using 0.25 % trypsin, the cell lines were grown at 37 °C in the presence of 5 % CO_2_ and 95 % air and cultured in Dulbecco’s modified Eagle’s medium (DMEM) mixed with 10 % fetal bovine serum (FBS), 75 µg/mL streptomycin, and 75 U/mL penicillin. In addition, the aseptic principle was strictly observed during the experimental operation. Before transfection, the cells were added to a plate with a density of about 2 × 10^5^ cells/cm^2^. Transfection was performed when 85 % confluence was reached. The plasmids (pCDNA3/rSK Ca2) used in this study were obtained from OriGene (USA). All the transfections were performed with Lipofectamine 2000 (Invitrogen, USA) following the manufacturer’s protocols. As described previously, stable expression of the SK2 gene was established in HEK293 cells (the cells are herein referred to as SK2 cells). Before the patch-clamp experiment, SK2 cells were seeded for about 24 h in the glass cover.

### Drugs and solutions

Trypsin, FBS, penicillin, streptomycin, and DMEM were all obtained from Gibco Invitrogen Corp. (USA). Bupivacaine, ropivacaine, and lidocaine were purchased from Sigma–Aldrich (USA). The Tyrode’s solution comprised the following: NaCl, 137mM; KCl, 5.4mM, MgCl_2_, 1.8mM; HEPES, 10mM; and glucose, 10mM; pH was maintained at 7.4 with NaOH. The pipette solution comprised the following: MgCl_2_, 1.15mM; potassium gluconate, 144mM; and CaCl_2_, 0.25mM/0.5mM/1.0mM); pH was maintained at 7.2 with KOH.

### Patch‐clamp experiments

All experiments were conducted using the whole-cell patch-clamp technique. The coverslip containing SK2 cells was placed under an inverted Olympus microscope (IX70, Japan) on the cell chamber. The solutions were added to the reservoirs from the superfusion system (DADVC-8PP, ALA SCIENTIFIC, USA). The DAD-VC systems have a Micromanifold comprising eight tubes of polyamide-coated quartz glass of 100 μm ID. The Micromanifold enables up to eight solutions from the reservoirs to flow into a small common space of less than 1 µL. The Micromanifold with a micromanipulator can be easily moved around the cell preparation and pointed at the target cell.

An EPC-10 amplifier (HEKA, Germany) was used for the whole-cell patch-clamp technique. A glass electrode with 1.2-mm outer diameter was pulled out with a microelectrode puller (P-97, SUTTER, USA) to achieve a resistance of 1.5–3.0 MΩ after adding the pipette solution. Under the microscope, SK2 cells with smooth cell membranes were picked up to record the currents. After gigaseal formation, negative pressure was introduced to break the SK2 cell membrane. Voltage stimulation and data recording were performed using the Pulse 8.0 software (HEKA, Germany). All experiments were performed at 36 °C. SK2 cells could produce stable currents at 0 mV. Therefore, currents at 0 mV were used for comparisons in the following experiments. SK2 cells were recorded for currents in three different phases: baseline, inhibition, and washout. The baseline phase involved the perfusion of SK2 cells with Tyrode’s solution. The inhibition phase involved the perfusion of SK2 cells with Tyrode’s solution containing LAs. The washout phase involved the replacement of LA-containing Tyrode’s solution with normal Tyrode’s solution. The currents recorded in the three phases were defined as Current_baseline_, Current_inhibition_, and Current_washout_. The normalization current was represented by Current_inhibition_/Current_baseline_. The normalization inhibition was calculated as (Current_baseline_ − Current_inhibition_ )/Current_baseline_.

### Statistical analysis

The SPSS software (version 19.0, IL, USA) was used for data analysis. The normality of data was tested using the Shapiro–Wilk test, and the normally distributed data were expressed as the mean ± standard deviation. Differences between the two groups were assessed using the Student *t* test, and ANOVA was used for comparisons of multiple groups. A *P* value < 0.05 indicated a statistically significant difference.

The relationship between local anesthetic concentration and its inhibitory effect on SK2 currents was fitted in a nonlinear fashion using GraphPad Prism 5.0 software (GraphPad, CA, USA). The equation was Y = Bottom + (Top − Bottom)/(1 + 10^((LogIC50−X)*HillSlope)^), where HillSlope represents the steepness of the family of curves, Top and Bottom represent plateaus in the units of the *y*-axis, X represents the logarithm of concentrations of LAs (0, 0.5, 1, 2, 2.5, and 3), and Y represents the normalization current. Normalization current was calculated as Current_inhibition_/Current_baseline_.

## Results

### Concentration–response relationship of bupivacaine, ropivacaine, and lidocaine on the inhibition of SK2 currents

**Fig. 1:**
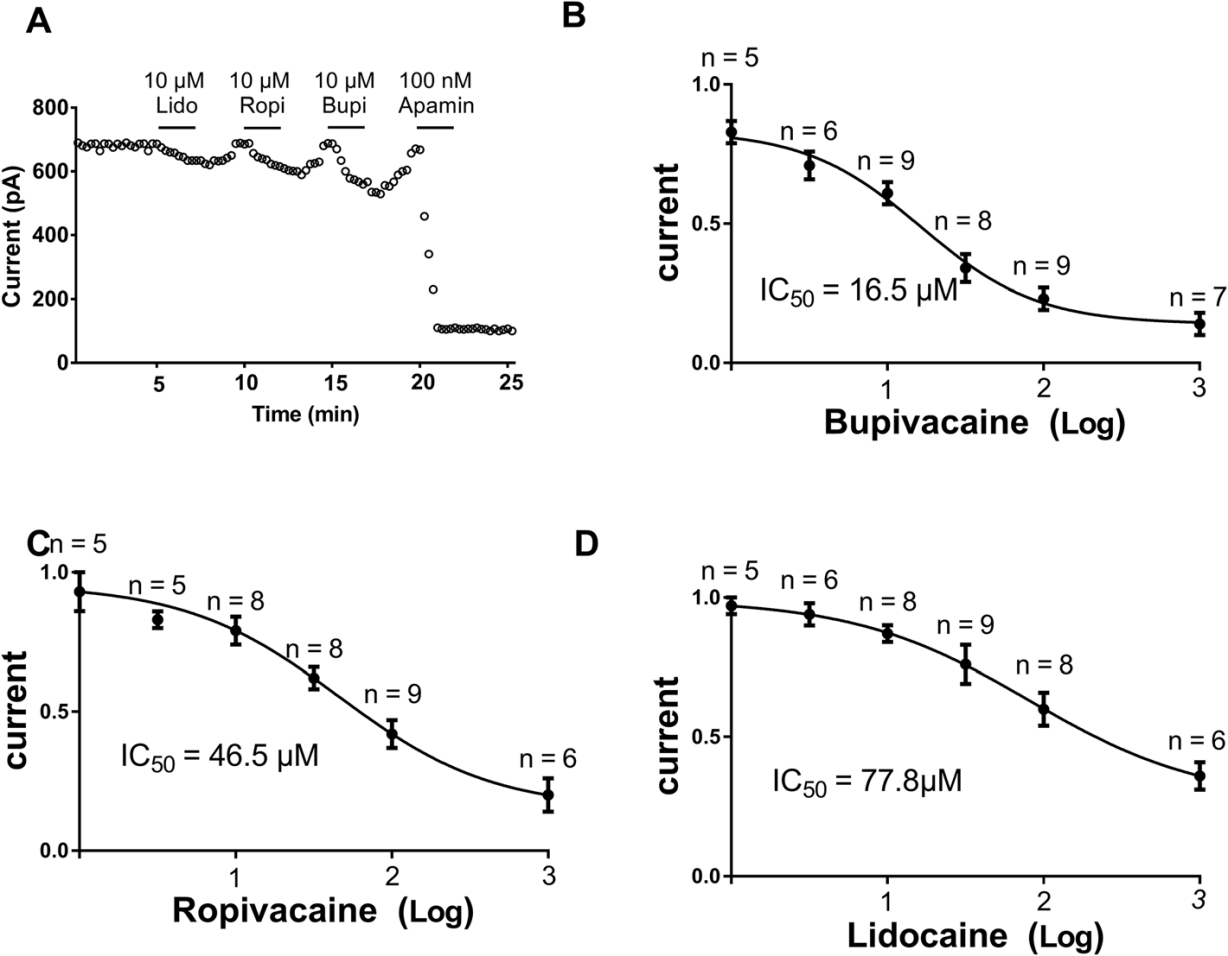
Concentration-dependent inhibitory effects of bupivacaine, ropivacaine, and lidocaine on SK2 currents.** a** The SK2 current was inhibited by local anesthetics and apamin in an SK2 cell. **b**, **c**, and **d** Dose-dependent effects of bupivacaine in terms of inhibiting SK2 currents (0 mV) were fitted to the Hill equation to obtain the IC50 value of bupivacaine, ropivacaine, and lidocaine, respectively. The equation was Y = Bottom + (Top − Bottom)/(1 + 10^((LogIC50-X)*HillSlope)^). The pipette solution contained 1μM free calcium

HEK 293 cells transfected with the SK2 gene (transfected cells were named SK2 cells) produced representative current tracings. The SK2 current was inhibited by local anesthetics in SK2 cells (Fig. 1 A). The half-maximal inhibitory concentration (IC50) value for bupivacaine was 16.5 µmol/L (95 % CI: 12.46–21.83; Fig. 1B). The IC50 value for ropivacaine and lidocaine was 46.5 µmol/L (95 % CI: 31.37–69.03) and 77.8 µmol/L (95 % CI: 55.66–108.7), respectively (Fig. 1 C and 1D).

### Inhibition of SK2 currents with LAs was reversible

**Fig. 2:**
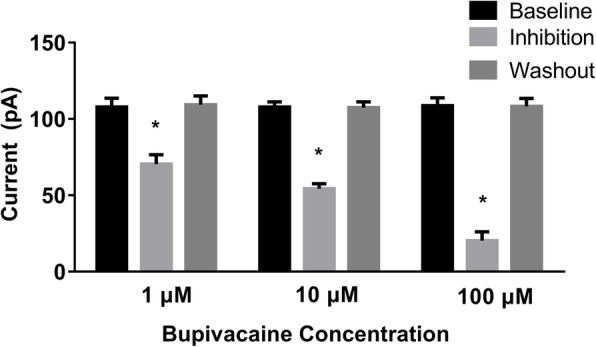
Inhibitory effect of bupivacaine on SK2 currents was reversible (*n* = 7 for each concentration).SK2 currents (0 mV) obtained at baseline and in inhibition and washout phases with exposure to 1μM, 10μM, and 100μM bupivacaine, respectively. Baseline: perfusion with Tyrode’s solution. Inhibition: perfusion with Tyrode’s solution containing bupivacaine. Washout: replacement of bupivacaine-containing Tyrode’s solution with normal Tyrode’s solution. Normalization inhibition was calculated as (Current_baseline_− Current_inhibition_)/Current_baseline_. The intrapipette free calcium concentration was 1μM. ^*^P < 0.05, compared with the baseline value

Next, the study explored whether the inhibitory effect of bupivacaine was reversible. In this part, SK2 currents from SK2 cells were recorded during exposure to 1µM, 10µM, and 100µM bupivacaine. Consequently, SK2 currents measured at 0-mV membrane potential were completely reversed to the baseline value after washout (*P >* 0.05; Fig. 2).

### Modulation of the inhibitory effect of bupivacaine by calcium concentration

**Fig. 3:**
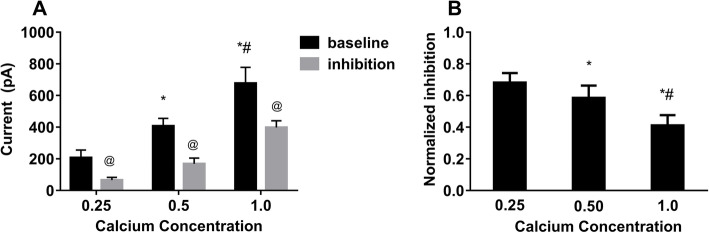
Effect of calcium concentration on the inhibitory effect of bupivacaine. **a** SK2 currents obtained at 0 mV in the presence of different concentrations of free calcium. **b** Degree of inhibition of SK2 currents (0 mV) by 10μM bupivacaine when the pipette solution contained different concentrations of free calcium. Baseline: perfusion with Tyrode’s solution. Inhibition: perfusion with Tyrode’s solution containing bupivacaine. Washout: replacement of bupivacaine-containing Tyrode’s solution with normal Tyrode’s solution. Normalization inhibition was calculated as (Current_baseline_ − Current_inhibition_)/Current_baseline_. ^*^P < 0.05, between 0.25μM group and 0.5μM group, ^#^*P *< 0.05, between 0.5μM group and 1.0μM group. ^@^*P *< 0.05, between inhibition value and baseline value

The opening probability of the SK2 channel is related to the intracellular calcium concentration. The present study tested whether the inhibitory effect of bupivacaine on SK2 currents were affected by calcium concentration. Figure 3 A shows traces of the SK2 currents induced in the presence of intracellular free calcium concentrations of 0.25, 0.50, and 1.0µM. The results showed an increase in the SK2 currents, as the calcium concentration increased and reached 1.0µM (*P* < 0.05). Figure 3B shows that bupivacaine inhibited SK2 currents to different extents in the presence of different intrapipette concentrations of free calcium. The results showed that SK2 currents were inhibited the least at a calcium concentration of 1.0µM (*P* < 0.05).

## Discussion

The results of this study were as follows: (1) Bupivacaine could reversibly inhibit the SK2 channel in a dose-dependent manner. (2) The IC50 value of bupivacaine, ropivacaine, and lidocaine for inhibiting SK2 was 16.5µM, 46.5µM, and 77.8µM, respectively. (3) The intracellular calcium concentration could influence the inhibitory effect of bupivacaine on SK2 currents.

After transfection with the SK2 gene, SK2 cells produced stable SK2 currents, which could be inhibited by apamin. Therefore, the SK2 current is also called apamin-sensitive current [18]. Since the opening of the SK2 channel was mainly dependent on the calcium concentration, the SK2 currents were recorded at 0 mV voltage. The intracellular free calcium concentration was controlled using the electrode solution, and the extracellular buffer did not contain free calcium.

Bupivacaine cardiotoxicity results from the blockade of a wide range of myocardial ion channels, the most important being the sodium channel [6, 7]. In this study, the whole-cell patch-clamp technique was used to investigate the effects of LAs on SK2 currents, and the IC50 values of bupivacaine, ropivacaine, and lidocaine were measured. Bupivacaine ranked first in its potency of inhibiting SK2 currents, followed by ropivacaine and lidocaine. Interestingly, this order of potency was consistent with the order of LA cardiotoxicity. The maximum recommended clinical dose of bupivacaine was 175 mg. If a patient weighing 70 kg was given 175 mg bupivacaine (blood volume was about 7 % of the body weight); the bupivacaine plasma concentration could reach 104.2µM. If 175 mg bupivacaine reached the heart quickly, it would immediately cause cardiac arrest. The concentration of bupivacaine in the heart would be much higher than 104.2µM [19]. Therefore, theoretically, the concentration of bupivacaine in the heart can reach an IC50 value of 16.5µM.

Martín et al. [20] examined the inhibitory effect of bupivacaine on large-conductance calcium-activated potassium channels in smooth muscle cells of the human umbilical artery. In his study, bupivacaine could block these potassium channels. Also, Sbarbaro et al [21]. found that lidocaine could block SK2 currents in nerve cells. However, lidocaine blocked SK2 currents only when its concentration exceeded clinical concentrations. The blockade of SK2 channels by lidocaine is unlikely to cause clinical effects. However, the present study found that SK2 channels were very sensitive to bupivacaine and ropivacaine. The specific mechanism underlying this inhibition is still unclear. The inhibitory effect of bupivacaine on the SK2 channel could affect several physiological functions and hence should be taken into account and considered as bupivacaine cardiotoxicity.

The present study also found that the intracellular calcium concentration could influence the inhibitory effect of bupivacaine on SK2 currents. SK2 channel proteins are coupled with calmodulin, and the binding of calcium with calmodulin alters the conformation and function of SK2 channels [22]. Studies have suggested that bupivacaine alters intracellular calcium concentrations, and hence bupivacaine is expected to indirectly regulate the SK2 channel. To eliminate the effects of this process, the intracellular calcium concentration in the pipette solution was controlled in this study. Consequently, this concentration-dependent inhibition of SK2 currents suggested that the concentration of intracellular free calcium contributed to bupivacaine cardiotoxicity.

Bupivacaine inhibits several ion currents in the heart (e.g., sodium channels, L-calcium channels, and potassium channels) [6, 7]. This study added SK2 channels to the list of ion channels affected by bupivacaine. SK2 channels caused arrhythmia depending on their expression levels in cardiomyocytes [23, 24]. These channels caused arrhythmia when the gene was expressed too much or too little in cardiomyocytes [25]. SK2 channels also participated in mitochondrial function [4, 26–28]. Therefore, the effects of SK2 channels on the action potential and mitochondrial function suggested that the blockade of SK2 channels was involved in bupivacaine cardiotoxicity. More experiments are needed to prove this hypothesis.

### Limitations

Under normal circumstances, the SK2 channel proteins are coupled with calmodulin. The binding of calcium with calmodulin affects the conformation and function of these channels [22]. However, this effect of calcium on SK2 channels was influenced because only the SK2 gene was transfected into HEK293 cells in the present study.

## Conclusions

The results of this study suggested the inhibitory effect of bupivacaine on SK2 channels. Future studies should explore the effects of SK2 channels on bupivacaine cardiotoxicity.

## Data Availability

The datasets used and analyzed in the current study are available from the corresponding author upon request.

## Abbreviations

SK2 channel: Small-conductance calcium-activated potassium type 2 channel
HEK293 cell: Human embryonic kidney 293 cell
IC50: Half-maximal inhibitory concentration
